# Pyrolytic Kinetics of Polystyrene Particle in Nitrogen Atmosphere: Particle Size Effects and Application of Distributed Activation Energy Method

**DOI:** 10.3390/polym12020421

**Published:** 2020-02-12

**Authors:** Lin Jiang, Xin-Rui Yang, Xu Gao, Qiang Xu, Oisik Das, Jin-Hua Sun, Manja Kitek Kuzman

**Affiliations:** 1 School of Mechanical Engineering, Nanjing University of Science and Technology, Nanjing 210094, China; ljiang@njust.edu.cn (L.J.); yxr1994@njust.edu.cn (X.-R.Y.); gaoxu@njust.edu.cn (X.G.); xuqiang@njust.edu.cn (Q.X.); 2 The Division of Material Science, Department of Engineering Sciences and Mathematics, Luleå University of Technology, 97187 Luleå, Sweden; oisik.das@ltu.se; 3 State key laboratory of Fire Science, University of Science and Technology of China, Hefei 264000, China; 4 Department of Wood Science and Technology, Biotechnical Faculty, University of Ljubljana, Jamnikarjeva 101, 1000 Ljubljana, Slovenia

**Keywords:** particle size, model free, model fitting, avrami–eroféev, DAEM

## Abstract

This work was motivated by a study of particle size effects on pyrolysis kinetics and models of polystyrene particle. Micro-size polystyrene particles with four different diameters, 5, 10, 15, and 50 µm, were selected as experimental materials. Activation energies were obtained by isoconversional methods, and pyrolysis model of each particle size and heating rate was examined through different reaction models by the Coats–Redfern method. To identify the controlling model, the Avrami–Eroféev model was identified as the controlling pyrolysis model for polystyrene pyrolysis. Accommodation function effect was employed to modify the Avrami–Eroféev model. The model was then modified to *f*(*α*) = *nα*^0.39*n* − 1.15^(1 − *α*)[−ln(1 − *α*)]^1 − 1/*n*^, by which the polystyrene pyrolysis with different particle sizes can be well explained. It was found that the reaction model cannot be influenced by particle geometric dimension. The reaction rate can be changed because the specific surface area will decrease with particle diameter. To separate each step reaction and identify their distributions to kinetics, distributed activation energy method was introduced to calculate the weight factor and kinetic triplets. Results showed that particle size has big impacts on both first and second step reactions. Smaller size particle can accelerate the process of pyrolysis reaction. Finally, sensitivity analysis was brought to check the sensitivity and weight of each parameter in the model.

## 1. Introduction

To meet the needs of of society, various kinds of advanced materials with different functions have been invented and updated greatly. In the ultrafine materials research area, researchers have tried to generate particles with even smaller diameters. After a normal particle is processed by ultrafine technology, particles will own some unique characteristics, including large specific surface area and high chemical activity. The peculiar physical and chemical characteristics make ultrafine particles the focus of advanced materials nowadays. During the processes of particles’ industrial manufacture, storage, and transportation, particles with different sizes behave differently when considering their safety concerns. Therefore, particle size effects are essential influence factors needed to be considered when researchers explore particle thermal safety problems. The chemical kinetics and reaction model can be greatly influenced by particle size [1]. After the block is processed by ultrafine processing technology, particle specific surface area can be greatly increased, which can influence combustible pyrolysis and reaction rates when heating, and even the reaction model and products can be changed [2,3].

So, the work reported here was motivated by a study of particle size effects on pyrolysis behavior, chemical kinetics, and reaction model when surrounded with heating. Micro-size polystyrene particles with four different diameters were selected as typical particle materials. Activation energies were obtained by several different isoconversional methods. The pyrolysis model of each particle size and heating rate was examined by nineteen different reaction model candidates by the Coats–Redfern methods, among which the three best models were then selected, and the reaction model function was then reconstructed by selected models. The particle size effects on kinetics and reaction model could be concluded. To separate step reactions from whole reaction and identify their distributions to kinetics, a distributed activation energy method was then introduced to calculate the weight factor and kinetic triplets.

## 2. Literature Review

Polystyrene is a commonly-used polymer material in daily life, which is usually employed as thermal insulation materials in extruded or expandable formation, whose kinetics and reaction mechanism have been studied. Jiao et al. studied the kinetics and volatile products of expandable polystyrene and extruded polystyrene with TGA and TGA-MS-FTIR, respectively. They found that the activation energies with conversions of expandable polystyrene are a little higher than extruded polystyrene, which means expandable polystyrene is a little more stable than the extruded one. During the pyrolysis process, small molecules including CO, C_2_H_3_, C_2_H_5_, and phenyl were detected [4]. After this, Jiao and Sun explored the reaction mechanism of polystyrene during the pyrolysis process. It was found that two pyrolysis reactions exist during the whole heating process. One is the small pyrolysis of styrene monomers around 275 °C, and the other is breakage of the main chain and large amounts of styrene generation around 430 °C [5]. Cheng et al. compared the thermal degradation behaviors of micron polymethyl methacrylate (PMMA) and polystyrene (PS) by a traditional kinetics method. They found that the particle size diameters can result in the decrease of activation energies, but have no obvious influence on pre-exponential factors [6]. Other researchers have conducted related studies about particle size effects on material pyrolysis behavior. Shen et al. [7] investigated the wood particle size effects on the yield of bio-oil production. Results showed that the yield of bio-oil production can decrease with the particle size increasing, among which the light bio-oil fractions increased and the heavy bio-oil decreased. Marcilla et al. [8] tested different sizes milled powders of almond shells and olive stones. They found that the milling process can provoke the structure damage of both biomasses, and thus cause the difference in thermal behavior. Also, the milling process may cause the increase of mineral substance. Blasi [9] investigated the particle size and heating rate effects on cellulose pyrolysis by means of a computational model. Three main regimes of particle sizes were found to control pyrolysis processes, including thermally thick, thermally thin, and pure kinetic control, which were adjudged by particle size and heating rate conditions. Hanson [10] studied particle size effects on pyrolysis of coal, and found that a smaller particle was more likely to produce char residue larger than itself. For larger particle pyrolysis, it is more likely to produce a fragment. Yu et al. [11] ground the coal sample by a planetary ball mill, and the coal samples were classified into three groups according to different ground particle sizes. They found that particles with different sizes contain different carbon and ash contents, which is resulted by the characteristics of coal’s uneven texture and solidity. 

Most selected samples of previous pyrolysis studies relating particle size effects were self-ground in the laboratory, among which biomass and coal were mostly employed. During the grinding process, it is hard to form particles with uniform shape and component, as these solids have uneven density and distribution. This can result in that the particles employed in thermal analysis experiments do not have uniform distribution, which can definitely cause thermal analysis profiles fluctuations and bad data repeatability. In this study, the polystyrene sample we used was produced by Suzhou Nanomicro Technology Co., Ltd. The particles were produced with uniform shape and diameter, the diameters of which were 5, 10, 15, and 50 µm. Uniform diameter can guarantee the veracity and reliability of experimental results. More details about experimental sample particle size can be found in Section 4. Most publications including the above reviewed ones preferred to employ a traditional kinetics method when dealing with polymer pyrolysis kinetics problems. However, for the case of polymer pyrolysis, there must be more than one reaction during the pyrolysis procedure. So, in this study, after the traditional kinetics analysis we will introduce distributed activation energy model to explore PS pyrolysis kinetics to distinguish the weight of each sub reaction.

## 3. Traditional Kinetic Methods

Thermogravimetric analysis (TGA) apparatus can heat the sample with a fixed heating rate and gas flow to blow off the volatiles, and record the instant mass loss. The mass conversion at a certain time can be calculated by instant mass loss divided by total mass loss. The pyrolysis reaction can be expressed by the arithmetic product of two functions, including reaction rate constant and reaction model,

d*α*/d*t* = *A*exp[−*E_a_*/(*RT*)]*f*(*α*) (1)

where *A*, *E_a_*, and *R* are the pre-exponential factor, the apparent activation energy, and the gas constant, respectively. By TGA testing technique and kinetics calculation methods, the kinetic details can be obtained by measurement and parameterization. After processing natural logarithm to both sides of Equation (1) and then integrating, the reaction rate can yield to

(2) $$g(\alpha )=\frac{A}{\beta }\int_{T_{0}}^{T}\exp(-\Delta E_{a}/RT)dT$$

in which the temperature part has no analytical solution. β means heating rate and equals to dT/dt. Many researchers have tried to solve the integration with reasonable approximations, commonly used methods like KAS [12,13], FWO [14,15], and Tang et al. [16,17] methods, among which different approximation solutions were employed to Equation (2) as listed in Table 1. 

Solved by numerical integration, kinetics parameters can be calculated more accurately with appropriate approximations. Vyazovkin et al. [18,19,20] developed an advanced isoconversional method which contains the temperature integration.

(3) $$I(E_{\alpha },T_{\alpha })=\int_{0}^{T_{\alpha }}\exp(\frac{-E_{a}}{RT})dT$$

(4) $$I=\frac{E_{a}}{R}p(x)$$

Then the Vyazovkin method equation can be expressed as Equations (3) and (4), where *x* equals to *E_α_/RT*. At a certain conversional extent, the value of apparent activation can be identified by minimizing the following formula,

(5) $$\Omega (E_{a})=\sum_{i=1}^{n}\sum_{j\neq i}^{n}\frac{I(E_{a}{}_{,\alpha },T_{a,i})\beta_{j}}{I(E_{a}{}_{,\alpha },T_{a,j})\beta_{i}}$$

The temperature integration can be calculated after a series of transforms. Farjas and Roura [21] derived the six-order Padé approximation, which can give an absolute error less than 10^−16^ for *x* > 12

(6) $$p(x)\approx \frac{\exp(-x)}{x}\times (\frac{x^{5}+40x^{4}+552x^{3}+3168x^{2}+7092x+4320}{x^{6}+42x^{5}+630x^{4}+4200x^{3}+12600x^{2}+15120x+5040})$$

By Equations (3)–(6), for each conversion the minimization value can be obtained, by this method, a relative dependency between activation energy and conversion range can be obtained.

Model fitting method is a reaction model exploring method using well-known different theoretical reaction models to fit experimental *α*–*T* profiles, meanwhile for each model a set of activation energy and pre-exponential factor can be obtained. The Coats–Redfern method is one commonly used model-fitting method, which explores the asymptotic series expansion with the following formula,

(7) $$\ln\frac{g(\alpha )}{T^{2}}=\ln(\frac{AR}{\beta E_{a}}[1-(\frac{2RT^{*}}{E_{a}})])-\frac{E_{a}}{RT}$$

where *g*(*α*) is the integral form of the reaction model as shown in Table 1, and T^*^ is the average temperature during all the heating process. For each reaction model as listed in Table 1, plotting ln[*g*(*α*)/*T*^2^] vs. 1/*T* can obtain sets of activation energy and pre-exponential factor. The model which has the best linearity with experimental profile is considered as the real reaction model.

There are nineteen commonly used reaction models in a kinetics area [5,6]. Each model will be used to fit the experimental formation with the obtainment of activation energy and pre-exponential factor. Then according to the fitness of experimental data and theoretical model calculation, one correlation coefficient can be obtained. So, for all nineteen models, there must exist one maximum correlation coefficient. In previous studies, usually the model with the maximum coefficient is identified as the ideal reaction model. However, sometimes the model with the maximum coefficient may be not the real reaction model, which can be checked by model reconstruction with experimental data. So, this model reconstruction [22,23,24,25,26] should be further processed to check if the obtained model can fit experimental profile well, and which procedure is necessary, but is usually ignored in previous related literatures.

The compensation effects means that there must exist one relation between the kinetics parameters that the change of activation energy causing a linear variation of the natural logarithm of the pre-exponential factor. The change of activation energy can be caused by the heating rate or model selection; however, they must be limited to one reaction. When several models are used in the same heating rate, several sets of activation energies and pre-exponential factors can be obtained, then the kinetics compensation effects can be created. The compensation effects between kinetics parameters can be expressed by the following formula,

(8) $$\ln A_{i}=a+bE_{i}$$

where *i* means that the kinetic parameters are obtained from the *i*-th model, and parameters *a* and *b* are kinetics compensation parameters.

All models listed can be examined by Coats–Redfern method, by which nineteen corresponding sets of kinetics parameters can be obtained. Then the calculated activation energy and the pre-exponential factor can be used to evaluate the compensation effect formula parameters *a* and *b*. Based on the obtained compensation effects formula, the pre-exponential factor at each conversional extent can be evaluated according to the activation energies obtained by isoconversional methods.

## 4. Distributed Activation Energy Method

The kinetics methods introduced above belong to traditional kinetics methodology, which usually regards the pyrolysis reaction as one overall reaction, and the activation energy at a certain conversion extent is regarded as global activation energy. However, for polymer pyrolysis reaction, it is unreasonable to take one overall pyrolysis as one step reaction. Considering this reason, distributed activation energy method (DAEM) is adopted to separate the total reaction into several parallel reactions, which was originally adopted to separate the sub-reactions of biomass and coal [27,28,29,30]. The idea of distributed activation energy was firstly brought up by Vand [31], and then was developed to solve the pyrolysis problem of coal by Pitt [32].

DAEM assumes that the total reaction can consist of several parallel reaction groups. For each reaction group, it has its own sets of reactions on a molecular level. The decomposition reaction on molecular level can be expressed as,

(9) $$d\left(\frac{m_{i}\left(t\right)}{m_{i}*}\right)/dt=A_{i}\exp(\frac{-E_{i}}{RT})\left(\frac{m*-m_{i}\left(t\right)}{m_{i}*}\right)$$

where *i* means the *i_th_* molecular level reaction, *m_i_(t)* means the volatile mass fraction at time *t*, m_i_* means the total volatile mass fraction, *A_i_* and *E_i_* are the kinetic parameters for this reaction.

Integrating Equation (9) and assuming that the species *i* is one of the pool reaction group of component *j*, then we have the following expression of degradation of component *j*,

(10) $$\alpha_{j}=1-\int_{0}^{\infty }\exp[-\int_{T0}^{T}\frac{A_{j}}{\beta_{j}}\exp(-\frac{E_{i}}{RT})dT]f(E)dE$$

(11) $$\frac{d\alpha_{j}}{dT}=\int_{0}^{\infty }\frac{A_{j}}{\beta_{j}}\exp[-\frac{E_{i}}{RT}-\int_{T0}^{T}\frac{A_{j}}{\beta_{j}}\exp(-\frac{E_{i}}{RT})dT]f(E)dE$$

*a_j_* means the conversion of component *j*. *f*(*E*) means that the group reaction in component *j* follows the distribution functions *f*(*E*), among which Gaussian distribution function is the earliest and most extensive applied one. The Gaussian distribution can be expressed as

(12) $$f_{G}(E)=\frac{1}{\sigma \sqrt{2\pi }}\exp(-\frac{{\left(E-E_{0}\right)}^{2}}{2\sigma^{2}})$$

where the distribution has the center at *E*_0_ and the standard deviation *σ*. The random distribution is distributed symmetrically at the left and right sides of *E*_0_. For Gaussian distribution, the range between *E*_0_ − 1.5*σ* and *E*_0_ + 1.5*σ* covers 99.7% random distribution. In this study, we consider 60 times standard deviation, which means the integration of Gaussian distribution ranges from *E*_0_ − 30*σ* and *E*_0_ + 30*σ*. All equations about DAEM have temperature integration, which cannot be solved accurately in Equations (10)–(12). So, an approximation about temperature integration is also recommended here, here we calculate *p*(*x*) the same as Equation (6). By calculating the *j_th_* component DAEM mass loss rate, the overall reaction formula can be calculated as a linear reaction combination of all components,

(13) $$\alpha =\sum_{j=1}^{M}c_{j}\alpha_{j}$$

(14) $$d\alpha /dT=\sum_{j=1}^{M}c_{j}{\left(d\alpha /dT\right)}_{j}$$

where *c_j_* means a weight factor equaling to the amount of volatiles formed from the *j_th_* pseudo-component decomposition. It should be noted that Gaussian distribution is a symmetric distribution centered at *E*_0_ from the shape of the curve. The distributed activation energy assumed that the total pyrolysis reaction is made of multiple parallel reactions, which is a reasonable assumption for polymer degradation.

## 5. Experimental

**Figure 1:**
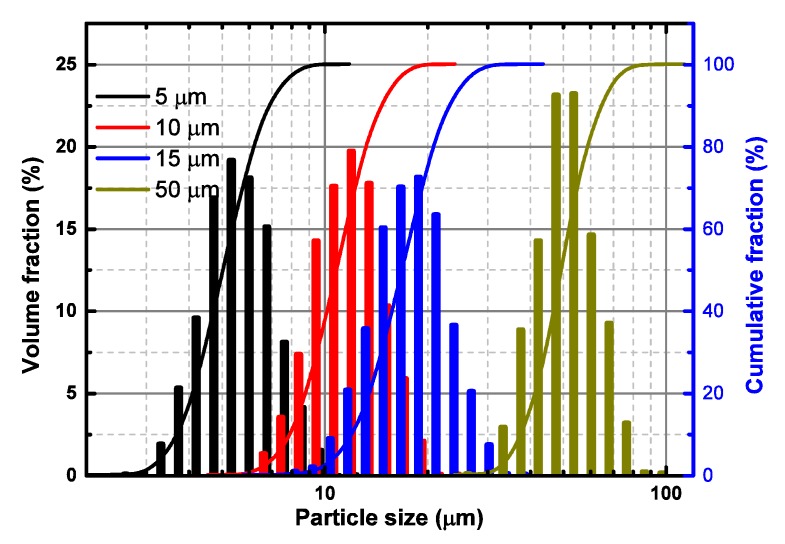
Particle size diameters of polystyrene particles with four different sizes, 5, 10, 15, and 50 µm.

Micro polystyrene particles were provided by Nano-Micro Technology Co., Ltd., Suzhou, China. Four available diameters, 5, 10, 15, and 50 µm, were selected. All particles showed uniform size according to the scanning electron microscopy examining figures (http://en.nanomicrotech.com/). Furthermore, the particle size was double checked by a Laser Diffraction Particle Size Analyzer, SALD-2300, produced by Shimadzu Corporation, Kyoto, Japan. Particle size was identified by the light intensity distribution pattern of scattered light that is irradiated from sample particle surface when laser lights radiate them. The particle size diameters for four particle sizes are shown in Figure 1.

The thermal degradation experiments were conducted on SDT Q600 instrument by TA Instruments (New Castle, USA). Experiments were performed in nitrogen atmosphere with 40 mL min^−1^ flow rate as purge gas and 20 mL min^−1^ as protective gas. Samples were heated in TGA with four heating rates, 3, 5, 7.5, and 10 K·min^−1^ from ambient temperature to 850 °C. An initial sample weight around 3 mg was guaranteed for all testing.

## 6. Results and Discussion

### 6.1. Pyrolytic Characteristics Observations

**Figure 2:**
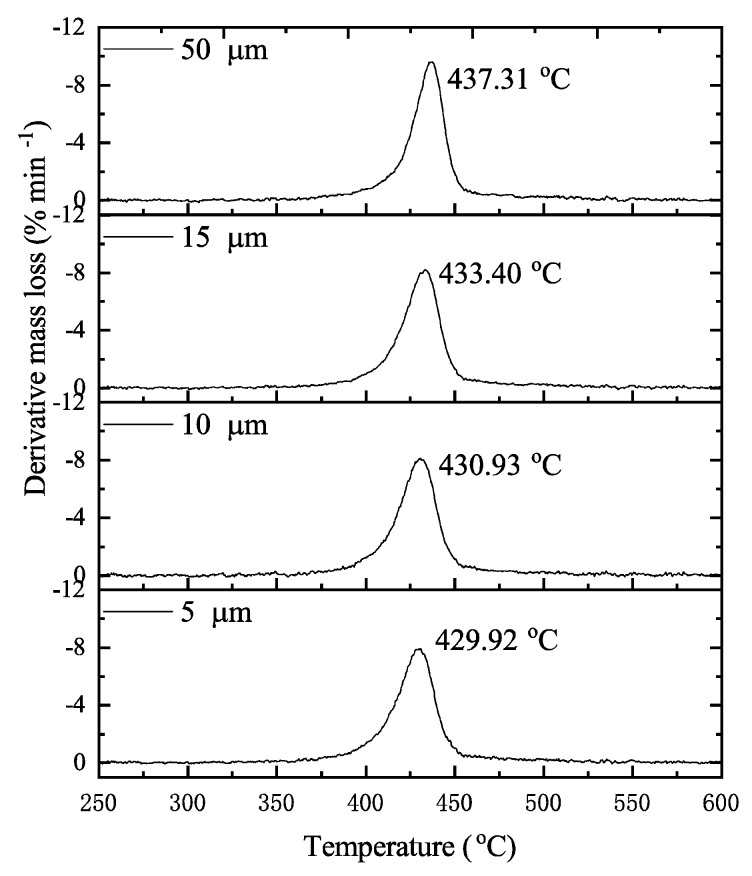
Differential thermogravimetry (DTG) rofiles of polystyrene pyrolysis in nitrogen atmosphere at 3 K·min^−1^ for 5, 10, 15, and 50 µm particle sizes.

Figure 2 shows the TGA and Differential thermogravimetry (DTG) profiles of polystyrene with four different sizes in nitrogen atmosphere. Detailed thermal pyrolysis temperatures are listed in Table 2. We can find that the pyrolysis profiles of polystyrene with different sizes show similar variations. The DTG curve shows an obvious single peak, which can be identified as a one step reaction. In nitrogen atmosphere, the percentage of heat loss keeps around 90.71 ± 0.80% constantly. From the TGA and DTG curves, we can find that sample sizes cannot cause the change of the reaction process or TGA profiles obviously.

For all the samples with different sizes, DTG curves show similar variations with one single peak, as the particles are produced from the same assignment. With the increase of particle size from 5 to 50 µm, the peak temperature increased monotonically. The 5 µm particle shows the minimum pyrolysis peak temperature and onset temperature, and 50 µm shows the maximum temperatures. For a polystyrene particle with a smaller diameter, it has a larger specific surface area, which means for the same sample masses, a smaller particle has more surface heated than a larger particle. For the TGA experiments in this study, we controlled all testing at the same weight at around 3 mg. Then for the 5 µm particle, its specific surface area is 10 times larger than 50 µm particle. Large specific surface area results in faster heat transfer and shorter time to trigger reaction.

### 6.2. Kinetics Parameters

**Figure 3:**
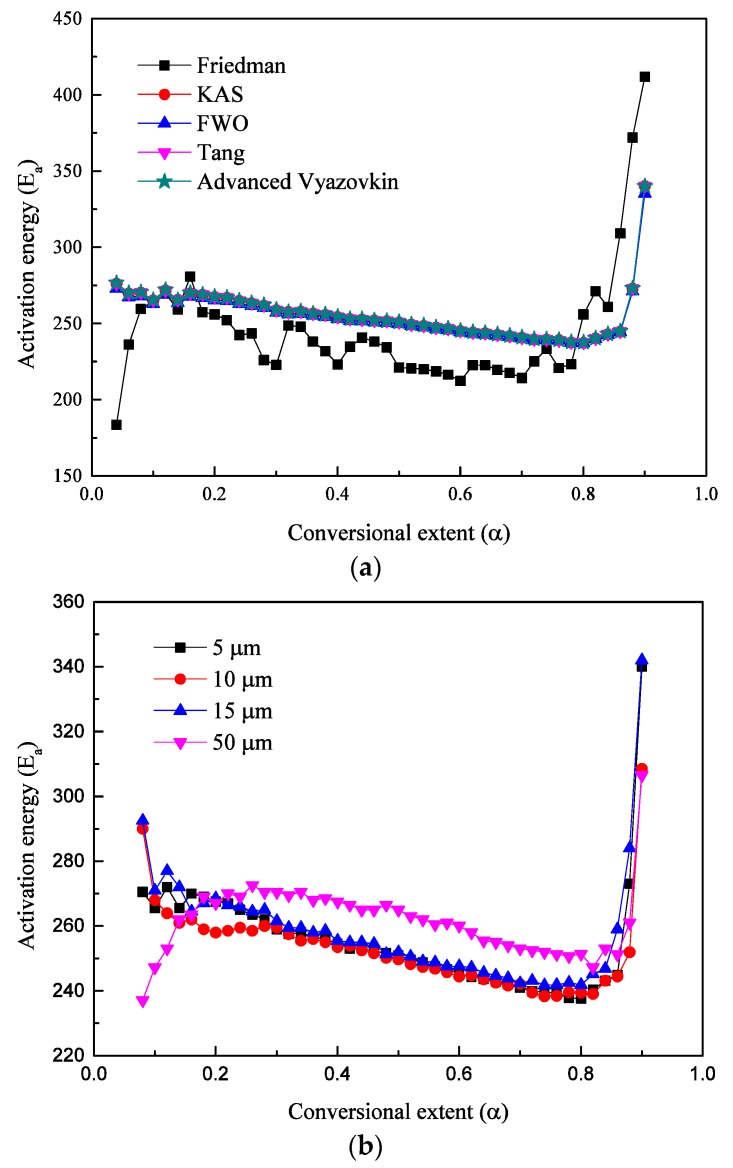
(**a**) Dependencies of the activation energy on extent of polystyrene conversion determined by five iso-conversional methods including KAS, FWO, Tang, Friedman, and advanced Vyazovkin methods. (**b**) Dependencies of the activation energy on conversional extent of four different size polystyrene determined by Vyazovkin methods.

The activation energies of polystyrene with four different sample sizes were calculated by five different commonly used isoconversional methods. Then, the dependences of activation energies on conversional extent for different calculated methods can be obtained. Figure 3a shows the activation energy calculation results based on different calculation methods. Five curves show the same variation with increasing conversional extent while Friedman results showed different variation from four other methods. The main reason that caused the deviation by the Friedman method with others is data noise brought during data differential process by total mass to use d*α*/d*t*, while the other four methods do not need a derivation step [33,34,35]. So FWO, KAS, Tang et al., and advanced Vyazovkin methods show almost the same calculation values, which proved the accuracy of the method calculation.

Figure 3b shows the dependencies of activation energies on conversional extent for four different polystyrene particle sizes. The activation energy results were calculated by the advanced Vyazovkin method. The advanced isoconversional method developed by Vyazovkin is a commonly used thermal kinetics method, which excluded the influences of reaction model and needs for differential data to obtain activation energies. From Figure 3b, we can find that the variation tendencies are the same. During the conversional extent 0–0.2, the activation energies fluctuate significantly because a small amount of styrene molecules pyrolyzes and escapes from the main chain. During the conversional extent 0.2–0.85, four size samples show the same variation tendencies. With the increase of conversional extent, the activation energies of all four samples decrease almost linearly, which stage corresponds to the pyrolysis of polystyrene main body. When *α* > 0.85, the activation energies increase rapidly with the increase of conversional extent. During this extent, the mass loss is mainly composed by polystyrene residue, which is hard to pyrolyze continuously and results in a rapid increase of activation energy.

During the main pyrolysis stage, with the increase of conversional extent, activation energies decrease slowly and linearly for all four sizes of samples. The activation energies of 5 and 10 µm are very close to each other for each conversional extent, both of which are smaller than activation energies of 15 µm particle size. The 50 µm size particle shows the maximum activation energies compared with another three sizes, which means that the reaction of 50 µm is the hardest to trigger. This difference on kinetics is mainly caused by their different specific surface area. For all four samples, 50 µm particle sample has the smallest specific surface area, therefore it has the maximum activation energies. The specific surface area of 5 µm particle size is 10 times than 50 µm particle size.

### 6.3. Model Fitting Method and Compensation Effects

By the isoconversional method calculation, we learned that the main pyrolysis stage (*a* = 0.2–0.85) of four sample sizes has constant activation energies where one existing reaction model may fit well. Isoconversional methods can only calculate the activation energies at a certain conversional extent, but fail to obtain the reaction model. With employment of the Coats–Redfern method, experimental data for four particle sizes can fit with all nineteen models. Then for each tested model, one set of activation energy and pre-exponential factor can be obtained. Three models with best linear coefficients for four sample sizes and heating rates are selected to list in Table 3, considering the linearity coefficient and activation energy appropriateness.

From the kinetics calculation results listed in Table 3, we can see that the kinetics triplet calculations are greatly dependable on the model selection. The activation energies calculated by Model 13 are around 225 kJ·mol^−1^, while for Model 8, the calculation result is around 463 kJ·mol^−1^. From Table 3, we can find that for all cases of each particle size and heating rate, the best three models are the same, i.e., first-order model (F1), Avrami–Eroféev (A3/2), and Avrami–Eroféev (A2). All three models show good linearity, larger than 0.98. However, the A3/2 and A2 models are more reasonable than the F1 model because the activation energies obtained by Avrami–Eroféev are closer to the results by isoconversional methods. Also, the experimental *f*(*α*) shows an increase first then decrease variation, whose variation tendency only fits the Avrami–Eroféev model. Although the dimensional diffusion model has the similar variation, its magnitude is too small to fit with experimental results.

Calculation of activation energy at each conversional extent allows the reconstruction of the pyrolysis model, which acquires pre-exponential knowledge in advance. For one fixed reaction at one known heating rate, the activation energies have a linear relation with natural logarithm of the pre-exponential factor called compensation effect, which can be expressed as ln*A_j_* = *a* + *bE_j_*, where *a* and *b* are constants for one reaction, *a* = ln *k_iso_* and *b* = 1/*RT_iso_*. *k_iso_* is called artificial isokinetic rate and *T_iso_* is defined as artificial isokinetic temperature. The subject *j* means the selected model. If the model we employed in calculation is not appropriately hypothesized, then the kinetic parameter artificial isokinetic temperature may locate out of the experimental temperature.

**Figure 4:**
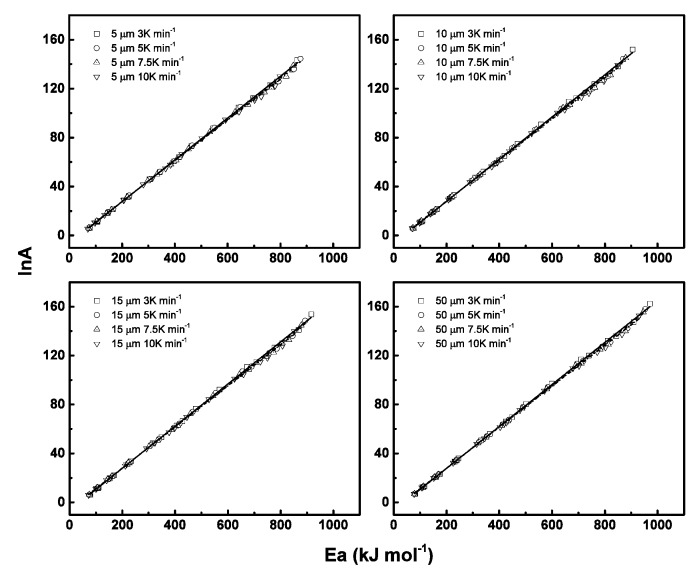
The isokinetic relationships (ln*A* vs. *E_a_*) obtained during degradation process using Coats–Redfern method for different particle sizes and heating rates.

For each model, one set of kinetic parameters can be calculated. Then all the kinetics parameters can be used for modelling compensation effects, as listed in Table 4. Results showed that all the heating rates for each particle size have good linearity, as shown in Figure 4, which allows for the prediction of the pre-exponential factor at each conversional extent.

### 6.4. Numerical Reconstruction

In Section 6.2, the activation energies at each conversional extent were obtained by isoconversional methods. Then, nineteen models were checked by the Coats–Redfern method to obtain a reasonable model describing polystyrene particle pyrolysis for cases of four different particle sizes. Avrami–Eroféev models (both A3/2 and A2) showed high linearity to the fitting with experimental profiles. Based on kinetic triplet results by different models, compensation effects could be employed to create numerical connection between activation energies and the pre-exponential factors, by which the pre-exponential factor at each conversional extent can also be clear. Based on the obtained pre-exponential factor on conversional extent, the calculated reaction model function can be obtained and compared with the theoretical reaction model function to examine the validity of the reaction model.

For all nineteen models, only the Avrami–Eroféev model can fit with experimental data during all conversional ranges; however, the results are still unsatisfactory to fit all heating rates well. This is because the most universally employed model in thermal kinetics is not applicable for reactions in/on media that are solid or porous structured [36]. So, when the pyrolysis kinetics are being described and refitted accurately, one accommodation function should be introduced to modify the model based on its known function. The real reaction model can be calculated by the arithmetic products of two functions, one is the accommodation function which can be expressed by *α^m^*, and the other is a classical reaction model. The new kinetics model after modification can be expressed by

*f*(*α*) = *nα^m^*(1 − *α*)[−ln(1 − *α*)]^1 − 1/*n*^ (15)

**Figure 5:**
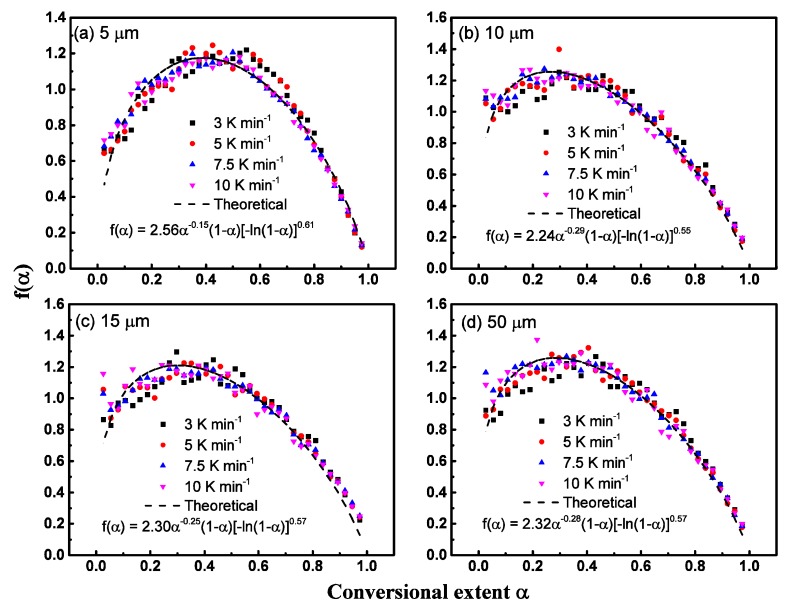
The experimental kinetics function *f*(*α*) reconstructed from isoconversional kinetic method of polystyrene pyrolysis for 3, 5, 7.5, and 10 K·min^−1^ heating rates. The dash line means the reconstructed profile of modified Avrami–Eroféev reaction model.

Figure 5 shows the comparisons of experimental *f*(*α*) points during all conversional ranges with theoretical profiles based on Equation (15) for four particle sizes. Results show that the experimental and theoretical data can match reasonably well during all conversional extents with two parameters *m* and *n* to describe the reaction model.

By further processing experimental data of each heating rate, sixteen sets of *m* and *n* parameters are obtained. We find that there is a roughly linear relationship between all *m* and *n*, which can be described by *m* = 0.39*n* − 1.15 with *R*^2^ = 0.92. Then, the pyrolysis model function can be rewritten by

*f*(*α*) = *nα*^0.39*n* − 1.15^(1 − *α*)[−ln(1 − *α*)]^1 − 1/*n*^ (16)

**Figure 6:**
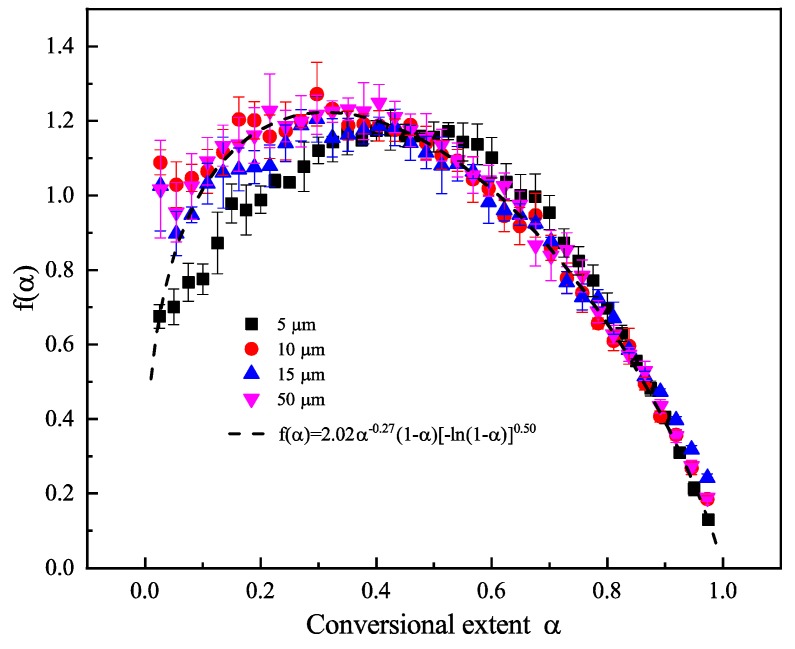
The experimental kinetics function *f*(*α*) reconstructed for 5, 10, 15, and 50 µm sample size.

As shown in Figure 6, four sample size experimental data were put together for model reconstruction since the reconstruction model lines in Figure 5 show similar variations. Results showed that for all four sample sizes, the reaction model can be described as *f*(*α*) = 2.02*α*^−0.27^(1 − *α*)[−ln(1 − *α*)]^0.50^. It can be concluded that the pyrolysis model, *f*(*α*), cannot be influenced by sample particle size because the geometric dimension cannot change the chemical reaction principles. Although the reaction model function *f*(*α*) cannot be influenced by particle size, the activation energies and reaction rate can be influenced greatly because the specific surface area can influence the heat transfer and evaporation rate of the particle surface.

It should be noted that in previous literatures about polymer pyrolysis model identification, it is far from enough that only linearity coefficients are obtained, by which the models are ranked. For each model will have its one linearity coefficient, and there must exist one model with the highest fitness; which however, does not mean that this model can describe the pyrolysis process well, especially when fitting with experimental data. Figure 5 and Figure 6 shows that the reconstructed model can describe the experimental well after modification, though the format of the final model shows difference with traditional nineteen models. We can also call the final reaction model an apparent model, which can be regarded as the combination of several step reaction models.

### 6.5. Step-Reaction Separation by Distributed Activation Energy Method

By traditional kinetics methods, we can only see that the activation energies are different for different sample size, while we cannot distinguish which step reaction makes the difference on pyrolysis kinetics. So, in this section, distributed activation energy method was employed to separate the step reaction from overall pyrolysis reaction, by which we can see the weight of step reaction on activation energy for different particle sizes. Details about the mechanism of DAEM have been introduced in Section 4, and the solution of DAEM equations was based on programming MATLAB to obtain the kinetics parameters. To improve the accuracy of kinetic results, experimental data of *α* and d*α*/d*t* was employed to fit by DAEM model at the same time, which was judged by getting the minimum value of squared sum residuals (SSR), which can be expressed by

(17) $$SSR=\sum_{n=1}^{e}\sum_{m=1}^{f}\{{\left[\alpha_{num}\left(T_{k}\right)-\alpha_{exp}\left(T_{k}\right)\right]}^{2}+{\left[{\left(\frac{d\alpha }{dT_{k}}\right)}_{num}-{\left(\frac{d\alpha }{dT_{k}}\right)}_{exp}\right]}^{2}\}$$

where *e* and *f* mean all heating rates and selected experimental data points. The subscripts num and exp mean the numerical DAEM model and experimental data, respectively.

For PS pyrolysis in nitrogen, the pyrolysis mechanism has been explored a lot. It is generally acknowledged that the pyrolysis process can be divided into two steps. The first step is the pyrolysis of the main PS structure with a generation of large volatile molecules, during which the structure will show a large mass loss. The second step is the generation of single molecule styrene mainly from the large molecule and a little bit from the residual body. During the DAEM calculation, we hypothesize that PS pyrolysis process includes two reaction steps. Equations (9)–(14) were solved based on genetic algorithm (GA) in MATLAB. GA is an advanced algorithm based on Darwin’s evolution theory, searching the best fitness in solving a high-dimensional optimization problem. For each new generation, GA will generate a certain amount of individuals randomly and simultaneously, among which each individual will be employed to fit with experimental data with fitness obtained. The individual with best fitness will be adopted as a parent to produce next generation. During producing, each generation process, selection, interaction, cross, and variation are all considered. Finally, one individual with best fitness is identified as the final parameters.

**Figure 7:**
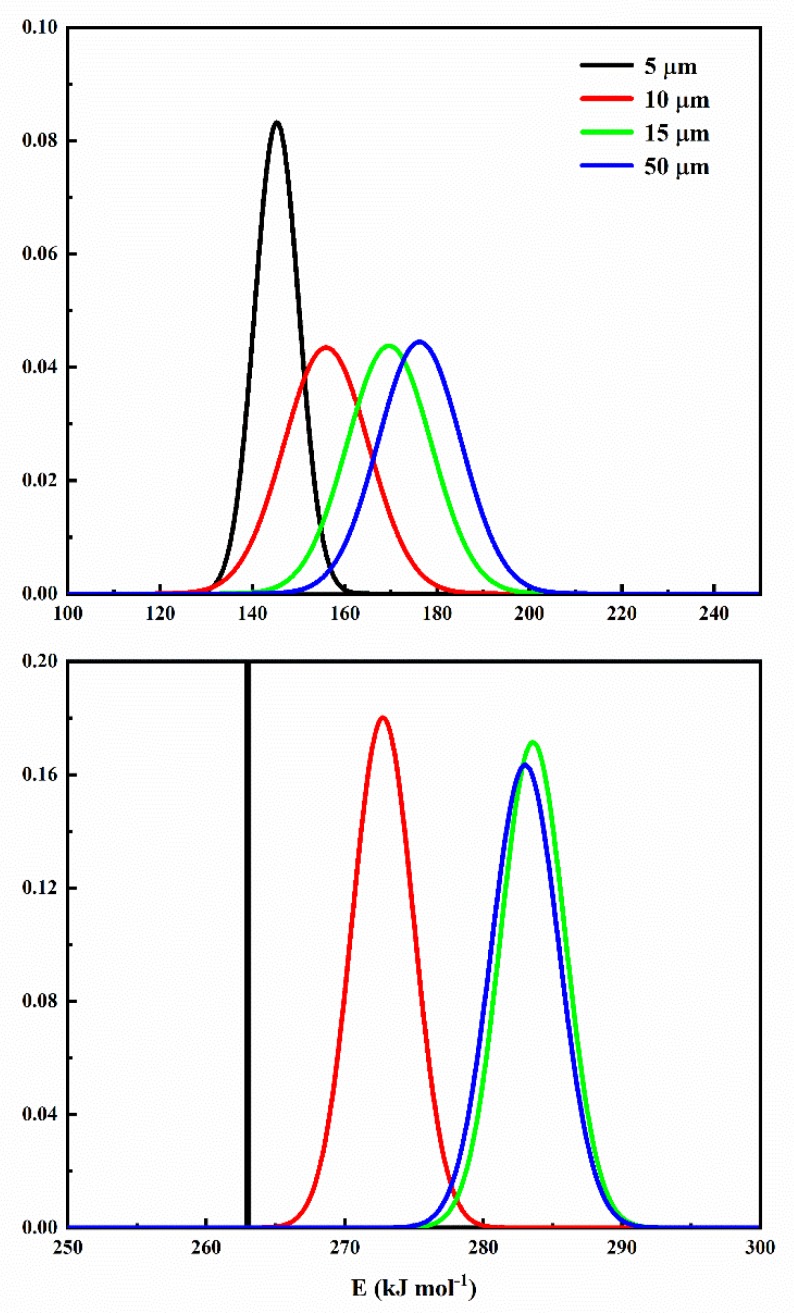
Activation energy distribution in distributed activation energy distribution method with Gaussian distribution for four particle sizes.

The aforementioned two-pseudo-component pyrolysis mechanism was employed during DAEM, and the searching ranges for four parameters, natural logarithm of pre-exponential factor, standard derivation of Gaussian distribution, activation energy, and weight factor were 5–60, 0–15 kJ mol^−1^, 100–380 kJ mol^−1^, and 0–1, respectively. In each heating rate, 100 points with uniform intervals were selected from the original data during the 600–900 K temperature range. Table 5 shows the DAEM calculation parameters with best fitness for two component reactions hypothesis. Figure 7 shows the activation energy distributions for both step reactions. From Table 5 and Figure 7, we can find that the activation energy distributions of 5 µm is more concentrated than the other three particle sizes especially for the second step reaction at 260–290 kJ mol^-1^, which means the 5 µm particle is much easier to pyrolyze compared with other particles, and the first step reaction group is more concentrated. The centered activation energy increases with particle size increasing in both reaction processes, which is in accordance with the results by the isoconversional method. Obviously, the particle size effects on the second reaction are more obvious than the first step reaction.

**Figure 8:**
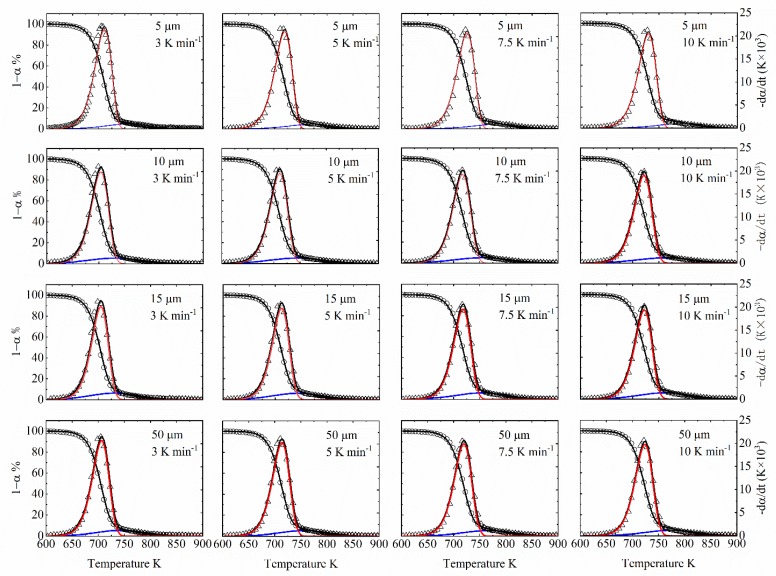
Comparison between DAEM calculation (solid lines, including *α* and d*α*/d*t* for overall reaction and step reactions) and experimental data (points, including *α* and d*α*/d*t*) for different particle size with different heating rates.

Figure 8 shows the experimental *α* and d*α*/d*t*, DAEM fitting *α* and d*α*/d*t*, and step reaction distributions. We can find that the experimental data and DAEM fitting can match each other reasonably well for all sixteen cases. And the mass loss by the first reaction occupies most of the reaction.

To quantitatively show the fitness between calculation and experimental, here we use Equation (18) to evaluate the fitness, and the higher result means better fitness, here we employ the weight coefficient as 0.5, Equation (18) can be expressed as

(18a) $$Fit_{\nu 1}=1-\sqrt{\sum_{m=1}^{b}{\left[{\left(\frac{d\alpha }{dT}\right)}_{num}-{\left(\frac{d\alpha }{dT}\right)}_{exp}\right]}^{2}/f}/{\left[{\left(\frac{d\alpha }{dT}\right)}_{exp}\right]}_{\max}$$

(18b) $$Fit_{\nu 2}=1-\sqrt{\sum_{m=1}^{b}{\left(\alpha_{num}-\alpha_{exp}\right)}^{2}/f}/{\left(\alpha_{exp}\right)}_{\max}$$

(18c) $$Fit_{\nu }=\left[\kappa Fit_{\nu 1}+\left(1-\kappa \right)Fit_{\nu 2}\right]\times 100\%$$

Table 6 shows the fitness results for different heating rates during DAEM fitting. We can see all fitness are larger than 98.5%, which proves the good performance of DAEM in TGA and DTG curve prediction.

### 6.6. Sensitivity Analysis of DAEM Parameters

**Figure 9:**
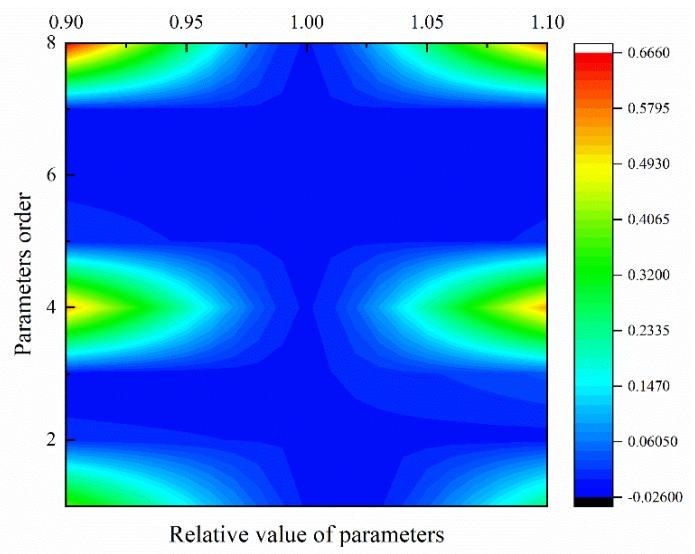
Sensitivity of eight DAEM parameters for 50 µm PS pyrolysis. This figure shows 50 µm PS particle case, and the sensitivity of other particles shows the same weight distribution.

After calculating the DAEM parameters of different particles, we also need to carry out the sensitivity analysis to judge which parameter is more important and sensitive. The method to check its sensitivity is to change the target parameter by a small value and remain the rest parameters unchanged. The variation range of parameter is very small, here we employ the range ±0.1. lg(ssr) to quantitatively judge the parameter sensitivity, where ssr is the SSR with changed parameter divided by the optimal SSR value. Parameters ln*A*_1_, *σ*, *E*_0,1_, *n*_1_, and ln*A*_2_, *σ*, *E*_1,2_, *n*_2_, are numbered as 1–8 as shown in y-axis of Figure 9, where x-axis means the relative changed value of parameters ranging from 0.9 to 1.1, y-axis means the order of eight parameters, and the color value in Figure 9 means lg(ssr). The blue color presents that the parameter is insensitive and accurate, while the red color means sensitive to the value change. So, during calculation, we should check the accuracy of these sensitive parameter to make sure its accuracy. Obviously, the pre-exponential factor and activation energy we obtained by DAEM methods are insensitive compared with weight factor and distribution factor, which means the result is reasonably dependable. Here in Figure 9, the data is 50 µm PS pyrolysis DAEM parameters. The parameters of other particle sizes show the same weight with 50 µm particle, so here we won’t discuss other particle size cases anymore.

## 7. Conclusion

Here we explore the particle size effects on pyrolysis of polystyrene from aspects of pyrolysis behavior, kinetics, reaction model, reconstruction, and validation. The final reaction model can provide scientific guidance to polymer pyrolysis modeling [22,23,24,25,26,27]. In this study, to explore the particle size effects on pyrolysis behavior, polystyrene particles with four different sizes, 5, 10, 15, and 50 µm, were selected to conduct a series of TG experiments. Isoconversional methods were employed to calculate kinetic parameters during all conversional extents. Results show that the temperature of the DTG curve peak will decrease first, then increase with particle size for the same heating rate, which may be caused by the competition of compactness and specific surface area effects. During the main pyrolysis stage, with the increase of conversional extent, activation energies decrease slowly and linearly for all four size samples. With the increase of particle size, the activation energies will increase for the same conversional extent, which means that the reaction of the largest particle is the hardest to trigger. The Avrami–Eroféev model was identified by the Coats–Redfern method as the controlling model during the polystyrene pyrolysis process. Considering the accommodation function of the reaction model, Avrami–Eroféev model was modified as *f*(*α*) = 2.02*α*^−0.27^(1−*α*)[−ln(1−*α*)]^0.50^, by which the polystyrene pyrolysis process can be well explained. To find the weight of each step reaction, the DAEM model was employed to separate the step reaction from overall reaction. Results showed that both step reactions can be largely influenced by particle size, especially for the second step. For the five µm particle, the activation energy distributions in both step reactions are more concentrated and forward, and its reaction is more uniform.

## Figures and Tables

**Table 1:** Three commonly used isoconversional methods for activation energy calculation.

Methods	Expression	Description
Flynn–Wall–Ozawa method	log*β* = log (*AE_a_*/*Rg*(*α*)) − 2.315 − 0.4567*E_a_*/*RT*	Modified general isoconversional equation by Doyle approximation.
Kissinger–Akahira–Sunose	ln(β/T^2^) = ln(*AR*/*E_a_g*(*a*)) − *E_a_/RT*	Modified general isoconversional equation by Coats-Redfern approximation.
Tang et al.	ln(*β*/*T*^1.894661^) = ln[*AE_a_/Rg*(*α*)] + 3.635041 − 1.894661ln*E_a_* − 1.001450*E_a_*/*RT*	Tang et al. proposed an improved approximation for temperature integral.

**Table 2:** Characteristic temperature *T*_0_, *T_p_* and *T_f_* for polystyrene pyrolysis determined from thermogravimetric analysis (TGA) profiles at different heating rates.

*β* (^o^C·min^−1^)	*T*_0_ (^o^C)	*T_p_* (^o^C)	*T_f_* (^o^C)	*α*_max_
5 µm				
3	367	430	535	91
5	351	438	545	92
7.5	369	443	528	91
10	349	447	530	92
10 µm				
3	378	431	572	92
5	378	437	531	91
7.5	381	458	528	90
10	379	460	534	91
15 µm				
3	368	433	524	90
5	369	440	534	91
7.5	386	446	529	90
10	382	449	529	91
50 µm				
3	375	437.31	537.50	90
5	381	444.23	534.04	90
7.5	385	451.87	530.90	89
10	359	455.82	535.81	90

**Table 3:** Activation energies, pre-exponential, and corresponding linearity coefficient calculated by Coats–Redfern method for the three best models.

	3 °C min^−1^				5 °C min^−1^				7.5 °C min^−1^				10 °C min^−1^			
	Model	ln*A*	*Ea*	*r* ^2^	Model	ln*A*	*Ea*	*r* ^2^	Model	ln*A*	*Ea*	*r* ^2^	Model	ln*A*	*Ea*	*r* ^2^
5 µm	8	73.17	462.33	0.998	8	73.54	466.64	0.999	8	70.47	451.10	0.999	8	66.29	426.76	0.998
	12	45.99	304.33	0.998	12	46.41	307.16	0.999	12	44.47	296.76	0.998	12	41.76	280.52	0.998
	13	32.32	225.33	0.998	13	32.75	227.42	0.999	13	31.39	219.59	0.998	13	29.41	207.40	0.998
10 µm	8	75.06	469.28	0.990	8	71.09	448.69	0.988	8	71.57	453.59	0.987	8	68.97	439.20	0.988
	12	47.26	308.99	0.990	12	44.76	295.22	0.987	12	45.22	298.44	0.986	12	43.56	288.83	0.988
	13	33.28	228.84	0.989	13	31.52	218.48	0.987	13	31.95	220.87	0.986	13	30.77	213.65	0.987
15 µm	8	76.54	478.30	0.993	8	74.02	466.03	0.993	8	72.31	458.14	0.992	8	69.84	444.63	0.992
	12	48.26	315.00	0.993	12	46.73	306.77	0.992	12	45.71	301.48	0.992	12	44.15	292.45	0.992
	13	34.03	233.35	0.992	13	33.00	227.15	0.992	13	32.32	223.15	0.992	13	31.22	216.36	0.992
50 µm	8	80.36	502.22	0.989	8	77.78	489.67	0.984	8	76.64	485.47	0.983	8	75.33	478.41	0.983
	12	50.81	330.93	0.987	12	49.24	322.52	0.984	12	48.61	319.68	0.982	12	47.82	314.95	0.982
	13	35.96	245.28	0.988	13	34.89	238.94	0.983	13	34.51	236.78	0.982	13	33.99	233.22	0.981

Models 8, 12, 13 means first order model, Avrami–Eroféev model (*n* = 1.5), and Avrami–Eroféev model (*n* = 2).

**Table 4:** The values of *k_iso_* and *T_iso_* by model fitting methods for pyrolysis of polystyrene particles with four sizes.

	3 K min^−1^		5 K min^−1^		7.5 K min^−1^		10 K min^−1^	
Particle Size	*k_iso_*	*T_iso_*	*k_iso_*	*T_iso_*	*k_iso_*	*T_iso_*	*k_iso_*	*T_iso_*
5 µm	0.001446	704.10	0.002360	711.74	0.003338	719.24	0.004233	722.76
10 µm	0.001407	697.77	0.002181	705.57	0.003241	712.49	0.004162	716.62
15 µm	0.001447	698.59	0.002301	706.27	0.003332	713.09	0.004265	717.20
50 µm	0.001482	701.24	0.00233	708.72	0.003393	715.68	0.004423	719.17

**Table 5:** Distributed activation energy method (DAEM) fitness for different particle size with different heating rates.

Component	Parameter	5 µm	10 µm	15 µm	50 µm
Component 1	ln*A*_1_	38.7179	40.8492	42.6370	42.4292
		0.0063	2.2146	2.3269	2.4410
	*E*_0,1_	262.9934	272.7594	283.5884	283.0256
	*n*_1_	0.9004	0.8426	0.8339	0.8613
Component 2	ln*A*_2_	16.3841	18.9673	21.2544	21.8706
		4.7951	9.1801	9.1128	8.9726
	*E*_0,2_	145.3102	155.9962	169.6331	176.2200
	*n*_2_	0.1153	0.1614	0.1744	0.1430

**Table 6:** DAEM fitness for different particle size with different heating rates.

Fitness				
Particle Size	3 K min^−1^	5 K min^−1^	7.5 K min^−1^	10 K min^−1^
5 µm	98.06	98.01	98.20	98.29
10 µm	98.39	98.77	98.77	98.78
15 µm	98.13	98.74	98.75	98.71
50 µm	98.52	98.72	98.70	98.79
